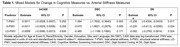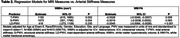# Total and Structural Carotid Artery Stiffness are related to Cognitive Decline and ADRD Pathology: The Multi‐Ethnic Study of Atherosclerosis (MESA)

**DOI:** 10.1002/alz.089226

**Published:** 2025-01-09

**Authors:** Theodore M DeConne, Jeremy R Williams, Ryan Pewowaruk, Claudia E. Korcarz, Jordan E. Tanley, Cynthia M. Carlsson, Susan R. Heckbert, Mohamad Habes, Ilya M. Nasrallah, Samuel N. Lockhart, José A. Luchsinger, Jingzhong Ding, Yongmei Liu, Kathleen M. Hayden, Adam D Gepner, Timothy M. Hughes

**Affiliations:** ^1^ Wake Forest University School of Medicine, Winston‐Salem, NC USA; ^2^ University of Wisconsin‐Madison School of Medicine and Public Health, Madison, WI USA; ^3^ Ryan Pewowaruk Research Consulting, Madison, WI USA; ^4^ University of Wisconsin School of Medicine and Public Health, Madison, WI USA; ^5^ Wisconsin Alzheimer’s Institute, University of Wisconsin School of Medicine and Public Health, Madison, WI USA; ^6^ University of Washington, Seattle, WA USA; ^7^ Glenn Biggs Institute for Alzheimer’s & Neurodegenerative Diseases, University of Texas Health Sciences Center at San Antonio, San Antonio, TX USA; ^8^ Department of Radiology, University of Pennsylvania, Philadelphia, PA USA; ^9^ Columbia University Irving Medical Center, New York, NY USA; ^10^ Wake Forest University School of Medicine, Winston Salem, NC USA

## Abstract

**Background:**

Stiffening of the large elastic arteries is an emerging age‐related risk factor for Alzheimer’s disease (AD) and related dementia (ADRD). Arterial stiffness is associated with pathological changes underlying AD/ADRD, and total arterial stiffness (T‐PWV) can be subdivided into two main mechanisms. Structural stiffening (S‐PWV) is due to intrinsic remodeling of the artery wall, and load‐dependent stiffening (LD‐PWV) is due to increased blood pressure without intrinsic changes to the artery wall. We hypothesized that greater structural stiffness (S‐PWV rather than LD‐PWV) would be associated with faster cognitive decline, and with increased white matter hyperintensity volume (WMHv) and decreased white matter fractional anisotropy (WMFA). Lower WMFA is interpreted as indicating poor WM integrity.

**Method:**

Multi‐Ethnic Study of Atherosclerosis (MESA) participants included in this analysis, represented four racial/ethnic groups that completed carotid arterial stiffness by duplex ultrasound (2010‐2012) from which pulse wave velocity was calculated, repeated cognitive assessments with the Cognitive Abilities Screening Instrument (CASI), Digit Symbol Coding (DSC), and Digit Span (DS) (2010‐12, 2016‐2019, 2019‐2021, Table 1), and 3T MRI (2016‐2021, Table 2). 3T MRI were acquired; T1, FLAIR and diffusion imaging sequences were acquired and processed to generate global WMHv and WMFA. Multivariable linear mixed and regression models related standardized arterial stiffness components to cognitive decline and neuroimaging and were adjusted for age, race and ethnicity, gender, education, site, and language.

**Result:**

Greater T‐PWV was significantly associated with faster declines in DSC and DS (Table 1), greater WMHv, and lower WMFA (Table 2), but not CASI. S‐PWV was significantly associated with faster declines in DSC and DS (Table 1) over a decade. Greater S‐PWV was also significantly associated with greater WMHv, but no WMFA differences (Table 2). Interestingly, LD‐PWV was not associated with changes in any measure of cognitive decline, and was associated with lower WMFA (Table 2).

**Conclusion:**

Higher structural stiffness of the carotid artery drives associations between total arterial stiffness with faster declines in cognitive function and with brain MRI findings. Interventions targeting arterial remodeling rather than blood pressure alone may be the next step in delaying age‐related cognitive decline and structural changes in the brain underlying ADRD.